# Pragmatic realism: towards a reconciliation of enactivism and realism

**DOI:** 10.1007/s11097-024-09959-w

**Published:** 2024-02-22

**Authors:** Catherine Legg, André Sant’Anna

**Affiliations:** 1 School of Humanities & Social Sciences, Deakin University, 221 Burwood Hwy, Burwood, 3125 VIC Australia; 2 Department of Philosophy, University of Geneva, 5 rue De-Candolle, Genève, 1211 Switzerland

**Keywords:** Pragmatism, Enactivism, Realism, Peirce

## Abstract

This paper addresses some apparent philosophical tensions between realism and enactivism by means of Charles Peirce’s pragmatism. Enactivism’s Mind-Life Continuity thesis has been taken to commit it to some form of anti-realist ‘world-construction’ which has been considered controversial. Accordingly, a new realist enactivism is proposed by Zahidi (*Phenomenology and the Cognitive Sciences,* *13*(3), 461–475, 2014), drawing on Ian Hacking’s ‘entity realism’, which places subjects in worlds comprised of the things that they can successfully manipulate. We review this attempt, and argue that whilst Zahidi rightly urges enactivists towards ‘internal realism’, he cannot sustain a non-negotiable aspect of realism that is crucial for scientific progress – the claim that multiple subjects inhabit *the same world*. We explore Peirce’s pragmatism as an alternative solution, foregrounding his distinction between existence and reality, and his inquiry-based account of cognition. These theoretical innovations, we argue, fruitfully generalize Zahidi’s *manipulation-based* enactivist realism to a richer, *inquiry-based* enactivist realism. We explore how this realism’s pan-species monism about truth encourages and supports the investigation of non-human animal cognition, and conclude by considering some implications of our discussion for long-standing realism debates within pragmatism.

## Introduction

This paper addresses some tensions arising between enactivist views of the mind and realist views of the world, and explores how a *pragmatic realism* inspired by Charles Peirce’s philosophy may coherently synthesise important ideas found in contemporary enactivism with some (but not all) tenets of traditional realism. Section 1 characterizes enactivism and discusses its apparent tensions with philosophical realism. Section 2 presents one recent reconciliation attempt – Karim Zahidi’s (2014) *manipulation-based realism* – arguing that despite some notable virtues it fails to convince. Sections 3 to 5 introduce elements from Peirce’s philosophy, in order to reconcile enactivism with the key realist intuition that multiple subjects inhabit the same world. We argue that Peirce’s distinction between ‘existence’ and ‘reality’ can integrate the enactivist claim that *reality* is in some sense mind-dependent with traditional realist claims that the same world *exists* for all subjects. We also consider how Peirce understands cognition as habits of forming, testing and correcting living expectations. The resulting *inquiry-based realism*, we argue, forms a more secure foundation for the kinds of scientific research that originally inspired enactivism.^1^ Moreover, its pan-species monism about truth encourages and supports the investigation of non-human animal cognition – the importance and value of which is increasingly being recognised. We conclude by discussing how our argument might help resolve longstanding perceived tensions between pragmatism and realism.

## Enactivism and Realism

Enactivism has influentially claimed that cognition is best understood as just another form of embodied action because, as articulated in a seminal text, “the two are not merely contingently linked in individuals; they have also evolved together” (Varela et al., 1991: 173). Following other commentators,^2^ we will refer to this as enactivism’s *Mind-Life Continuity Thesis*. This thesis emerged from a highly original intellectual framework forged from scientific biology, classical phenomenology, and Buddhist philosophy by pioneers Francisco Varela, Evan Thompson and Eleanor Rosch, drawing on work Varela had earlier done with Hubert Maturana (e.g. Maturana & Varela, 1980).

This radical reframing casts new light on a range of traditional philosophical problematics. A key example is the apparently common-sense metaphysical divide between knowing mind or ‘subject’ and known world or ‘object’. By contrast, enactivism understands cognition as depending “…upon the kinds of experience that come from having a body with various sensorimotor capacities, [which]…are themselves embedded in a more encompassing biological, psychological, and cultural context” (Varela et al., 1991: 173). Thus, contrary to traditional accounts (e.g. Fodor, 1983), cognition is no longer understood to simply record the state of an external world by means of internal representations. Rather, the subject *enacts its own set of meanings*, just as a living being grows and repairs its own body through *autopoiesis* (Varela et al., 1991; Thompson, 2010). But in so doing, the subject also *enacts its own environment* as having features of interest to itself, such as food or danger. This suggests that under enactivism, mind and world should be understood to be in fundamental ways co-determining (in Buddhist terms: codependently arising). Thus, Thompson writes:

…the object, in the precise sense of that which is given to and experienced by the subject, is conditioned by the mental activity of the subject…a cognitive being’s world – whatever that being is able to experience, know, and practically handle – is conditioned by that being’s form or structure (Thompson, 2005: 408).

This seems to imply a substantial shift in some mainstream philosophical conceptions of objective knowledge and scientific method.^3^ Certain enactivists have gone so far as to suggest that philosophers should newly integrate epistemology with ethics, such that “[o]ur manner of thinking can no longer be considered in isolation from our manner of being” (Vörös & Bitbol, 2017: 31, Varela, 1999).

These insights have often been taken to motivate a further, even more controversial claim: the *Anti-Representationalist Thesis*, which denies that basic aspects of cognition, such as perception, are contentful (see, e.g., Thompson, 2010; Rowlands, 2013, 2010; Hutto & Myin, 2017, 2012; Wheeler, 1997). According to this further thesis, the meanings that knowing subjects enact cannot be parsed semantically in the way that mainstream philosophers generally understand them – purely by reference to an independent set of objects and their properties (see, e.g., Varela et al., 1991: 172 − 83). Although enactivism has now developed into a number of distinct branches, not all of which hold the Anti-Representationalist thesis,^4^ by ‘enactivism’ we will mean views committed to both the Anti-Representationalist and Mind-Life Continuity theses.

These two theses call into question whether enactivists can coherently endorse *realism*. Although this term is extremely broad and contested, we believe it still has a powerful role to play in philosophy. Here we follow Peirce, who in 1911 wrote, “the word ‘reality’…is one of the words of whose meaning it is indispensible to have a perfectly distinct apprehension before drawing any conclusion, or forming any opinion, upon almost any philosophical subject” (Peirce manuscript R 852, cited in Lane 2018: 1). We acknowledge that when discussing such a fundamental notion, it is correspondingly difficult not to beg the question for one’s favored view. Therefore, our strategy will be to begin with the standard mainstream understanding of realism, to amend its definition as our unfolding discussion requires, and to thereby test how far enactivist views might be extended whilst still falling within some version of realism which still deserves the name.

For our mainstream understanding, we turn to *Realism and Anti-Realism* (Brock & Mares, 2007). This well-known book provides a useful definition of realism as encompassing an *existence* and an *independence* claim,^5^ as follows:

Realism about a particular domain is the conjunction of the following two theses: (i) there are facts or entities distinctive of that domain, and (ii) their existence and nature is in some important sense objective and mind-independent (Brock & Mares, 2007: 2).

The existence claim holds that a world exists, and the independence claim holds that that world’s nature is independent of any subject’s beliefs. Why make both claims? Broadly speaking, realists need the existence claim for the world to be present and substantive, and the independence claim for the world to be *stably knowable*. Of course, much will depend on exactly what is meant by ‘mind-independent’. Following Peirce, we take it to mean that whether a thing has or lacks certain properties does not depend on a subject’s thinking that it has or lacks those properties. (“That is real which has such and such characters, whether anybody thinks it to have these characters or not.” Peirce CP: 5.430).^6^ We can now see why enactivism is often associated with some form of anti-realism,^7^ through its claim that a knowing subject *constructs a world* as a unique coupling of their particular embodied context and their background understanding and habits, which then grounds their actions.

This notion of “world-construction” can be understood in at least two senses. The first, more metaphysical, sense is that subjects literally bring a world into being by cognizing it – without cognizing subjects, there would be nothing at all. The second, more epistemic, sense is that subjects ‘single out’ a world from some background that is in some sense present, but as yet uncognized. The first interpretation reads as a kind of subjective idealism, and thus as clearly anti-realist. It might be argued that the second sense need not have the same implications, as the ‘background’ (whatever it is) pre-exists the cognizer, albeit in uncognized form. Yet on the second sense, subjects with different embodiments will cognize different worlds, such that from within each subject’s perspective it will be impossible to tell how much any given feature is specific to their particular embodiment interacting with its particular environment. (This is of course the old philosophical problem of distinguishing ‘primary’ from ‘secondary’ qualities.) So although our second sense of world-construction may honor realism’s *existence claim*, it appears to violate its *independence claim*. We shall argue that it thereby multiplies worlds unduly. For insofar as subjects with different embodiments *seem to* interact with the same world, we apparently require an account of this. Consider for instance *color vision*. Although there are marked differences in how different species perceive colour, we don’t generally consider them to be perceiving different objects, but perceiving the same objects (fruit, flowers, their own species) differently.^8^

Some enactivists appear to explicitly embrace world-construction in the second sense. For instance, pioneers Varela, Thompson and Rosch urge us to reject “the idea of a world or environment with extrinsic, pre-given features that are recovered through a process of representation” (Varela et al., 1991: 137). Rather, we must understand our world as inseparable from our minds’ own processes of self-modification (Varela et al., 1991: 139) as the two codependently arise in a structural coupling (Varela et al., 1991: 205). They go so far as to diagnose philosophers’ reluctance to embrace this position as a “Cartesian anxiety” that we must find “a point where knowledge starts, is grounded, and rests, or we cannot escape some sort of darkness, chaos, and confusion” (Varela et al., 1991: 140). In later work, Thompson traces analogous links between enactivism and Merleau-Ponty’s phenomenological critique of ‘objectivism’:

For a bodily subject it is not possible to specify what the subject is in abstraction from the world, nor is it possible to specify what the world is in abstraction from the subject: “The world is inseparable from the subject, but from a subject which is nothing but a project of the world, and the subject is inseparable from the world, but from a world which the subject itself projects” (Merleau-Ponty 1962: 430, cited in Thompson, 2005: 410–411).

These are deep questions on which much more might be said. Yet we believe that embracing this kind of anti-realism is such a major philosophical step that before embarking on it, it would be well to ensure that it is entirely necessary. To be clear, we do not intend to argue that enactivists *should* commit to realism, but only that enactivism is *not at odds* with realism. So, while we think that there are advantages to the form of inquiry-based realism we will present—for instance, it allows us to explain interaction among species, and explore non-human cognition—we do not mean to settle the question of whether committing to realism is the only or the best strategy available to enactivists to deal with the issues we discuss.

## Zahidi’s enactivist entity realism

Zahidi (2014) presents a systematic argument that committing to anti-realism is not a necessary step for enactivists. He begins by observing that the mere fact that differing perspectives exist does not yet undermine the *independent reality* of what is shown from them, drawing a useful analogy to visual perspectives from different prison windows:

Consider two prisoners locked in different cells in different wings of a prison complex. Each cell looks out on a different courtyard…Neither of the two courtyards is universally accessible…But that does not mean that the two courtyards are not mind-independent or objective features of the prison complex (Zahidi, 2014: 466).

Zahidi rightly notes that “from the fact that an organism only represents certain features of the world, it does not follow that the organism represents the world as only containing those features” (Zahidi, 2014: 466). Nor, he later adds, does it follow from the fact that one sees things as being a certain way (X) that one sees them as *unable to be another way* (Y) (Zahidi, 2014: 470). But both entailments seem required in order for ‘world-construction’ in the second, epistemic, sense to undermine realism.

So how does Zahidi seek to reconcile realism and enactivism? Following Chemero (2011), he deploys philosopher of science Ian Hacking’s “entity realism”.^9^ Rather than defining realism in terms of whether certain theoretical terms *refer to things that exist*, Hacking claims that an entity such as an electron is real if it can be used to *intervene in other parts of reality* to produce measurable effects (Hacking, 1983: 262–3). Famously: “If you can spray them, they’re real” (Hacking, 1983: 24). Zahidi notes the essential link to *activity* in Hacking’s entity realism: “[b]y grounding the reality of theoretical entities not on their theoretical or representational usefulness, but on the fact that they can be manipulated, Hacking’s realism is rooted in human practical activity” (Zahidi, 2014: 470–1). He suggests that this makes entity realism a natural fit for enactivists’ analysis of cognition as *embodied action* (Varela et al., 1991), proposing the following definition of reality:

x is real for Y if and only if Y can manipulate x (Zahidi, 2014: 471).

Importantly, Zahidi here understands “manipulation” as performed by species or *types* of subjects, not single individuals. He thereby seeks to construct a shared world for each ‘manipulator-type’, stating: “The world for an organism type Y…consists simply of all entities that are real for Y” (2014: 471). Here the fact that members of any given species share evolutionary history and biological constitution explains how it is possible for them to inhabit ‘the same world’, through successfully manipulating their environment in similar ways (Zahidi, 2014: 471).

The way that this proposal generalizes across species appears to alleviate worries about our first, subjective idealist sense of enactivist ‘world-construction’, which contradicts Brock and Mares’ existence claim. But how does the proposal do on their independence claim? Not so well, we believe. For it renders mysterious how members of *different species* manage to interact, if they manipulate their environments very differently (a difference that Nagel famously noted regarding humans and bats: Nagel, 1974). Of course, *different* does not necessarily mean *distinct*. Zahidi does suggest that different species’ manipulable worlds often partially overlap, citing for instance predator and prey species (Zahidi, 2014: 473).

But not all species are linked through such interactions. Insofar as some lead completely separate lives, it appears that Zahidi must commit to multiple ‘species-worlds’. Zahidi argues that such multiplicity does not compromise his realism. He diagnoses the assumption that realists must restrict themselves to a single world as a kind of *scientific fundamentalism* which, he alleges, struggles to accommodate even core scientific contexts such as natural selection (Zahidi, 2014: 474). By contrast, he recommends a *pluralistic realistic ontological view*, noting that “[i]t is the equivocation of realism with universal fundamentalism that leads to anti-realism” (Zahidi, 2014: 474).

There is much to admire in Zahidi’s account. It astutely recognizes the invalidity of certain enactivist arguments for anti-realist claims, and synthesizes enactivism with a version of realism which is richly informed by scientific practice. Yet is it sufficiently realist to merit the name? We have seen that Zahidi commits to *type-mind-dependence* but not *token-mind-dependence*, by rendering the reality of worlds dependent on the manipulations of entire species. This takes an important step towards realism by denying subjective idealism. However, we can probe the account further in a number of directions. Firstly, it is unlikely to persuade the kinds of realists for whom Brock and Mares framed their definition of realism, who are convinced that the world exists *entirely* independently of the mind.^10^ Type-mind-dependence is still mind-dependence. Of course, as we have noted, Zahidi explicitly claims to provide a non-traditional realism. One might even seek to understand it as a kind of transcendental argument – a condition of possibility for successful interaction between different species. But we shall argue that the traditional realism which unreservedly posits a single mind-independent world is superior to Zahidi’s account insofar as it leaves room for science to *explain how interaction occurs between different species in the way that it does.*

We can illustrate our argument using Zahidi’s own prisoner analogy. In Zahidi’s realism, we can acknowledge that prisoners in cell 101 view one perspective of the world, while prisoners in cell 205 view another, and we can also allow that both perspectives form part of objective reality. But a further question cannot be explored: *why the prisoners in cells 101 and 205 see those particular perspectives*. To explore that question, we must ‘step back’ from the perspectives of both cells and posit an underlying ‘world’ in which they are both located – namely the jail itself, and its floorplan. This floorplan shows the directions from which the two prisoners are looking at the yard, and it thereby *explains why* each prisoner’s perspective contains and omits the features that it does contain and omit.^11^ Analogously, then, a genuine scientific realism must leave room for scientists to develop further explanations of the differing world-enactments of different species *by reference to one underlying reality in which they are differently embodied*.

A final concern with Zahidi’s account is that his pure manipulability account of reality accommodates only the world’s *reactive nature*, not its *intelligibility*. Both common sense and science extend beyond causally manipulating the world, to understanding and making successful general predictions about it, and our realism should reflect this.^12^ Thus, we shall now propose an alternative enactivist realism, based in Peirce’s pragmatism, focusing on two important points. The first is that Peirce repeatedly distinguished between *existence* – understood as the world’s materiality and causal efficacy – and *realit*y – understood as the general properties which structure worlds and render them intelligible. This distinction enables us to understand how enactivism may attribute Zahidi-style type-mind-dependence to a world’s reality, whilst not undermining commitment to its unitary existence. Our second point is that Peirce held an inquiry-based view of cognition which bears important similarities with the Mind-Life Continuity Thesis held by many enactivists, and also throws interesting new light on the relationship between reality and intelligibility.

## Pragmatic realism: existence and reality

Peirce famously believed that an adequate ontology must comprise a mix of fundamentally monadic, dyadic and triadic relations, as encapsulated in his three philosophical categories, or ‘modes of being’. *Category the first* consists in ‘pure presence’ – for instance, a sensation of red (Peirce 1903: 147). *Category the second* consists in ‘reaction’ – some kind of brute or “impositive” encounter between individuals, such as Hume’s famous banging of billiard balls causing motion (Peirce 1903: 160). This second category arguably corresponds to Zahidi’s manipulability, insofar as he follows Hacking in replacing representation with intervention as the basis for his realism (Zahidi, 2014: 471).^13^ But Peirce has one more category up his sleeve. *Category the third* consists in the ‘mediation’ of two things by a third. Here intelligibility emerges into Peirce’s ontology in cases where the ‘third’ thing is a concept.^14^ Imagine for instance that I accurately describe a patch of snow as *white*. Here the ‘first thing’ is me, the cognizer, the ‘second thing’ is the snow, the object of my cognition, and the ‘third thing’ is the concept of whiteness which I attribute to the snow. This application of the concept of whiteness enables me, the cognizer, to form future expectations about the snow – for instance that it can glare and hurt my eyes.

Peirce views these categories as all mutually irreducible and equally important. He aligns existence with Secondness – the world as directly encountered – and reality with Thirdness – the world as truly represented. Peirce is aware that his commitment to Thirdness as a mode of being over and above Secondness is a controversial position in modern Western philosophy, and accordingly he offers arguments for it. One argument that he offers in 1903 is very congenial to enactivism. He begins by asking *what is the use of thinking* to an organism located in an environment. He answers that without positing external “Reasonableness” (Thirdness) we would have no hope of gaining any knowledge from blind reactivity (Secondness):

…if the force of experience were mere blind compulsion…[we] never could make our thoughts conform to that mere Secondness. But the saving truth is that there is a Thirdness in experience, an element of Reasonableness to which we can train our own reason to conform more and more…therefore we need not wait until it is proved that there is a reason operative in experience to which our own can approximate. We should at once hope that it is so, since in that hope lies the only possibility of any knowledge (Peirce CP: 5.160).

An encounter between two billiard balls may be said to *exist*, as a particular interaction which happens in a certain spatiotemporal location, where it causes specific effects. But it is also *real* insofar as it shares characteristic features with similar interactions elsewhere, which are intelligible through the same general concepts, such as ‘force’ and ‘momentum’. Such general predications ground ongoing scientific practice, enabling scientists to successfully predict future events (Misak, 2004; Legg, 2001; Haack, 1992).

A crucial dimension of our pragmatist realism is that the identification of real regularities depends, ultimately, on the presence of *cognizers*. This follows from Peirce’s understanding of *truth* as “[t]he opinion which is fated to be ultimately agreed to by all who investigate” (Peirce CP: 5.408; Legg, 2014a; Misak, 2004; Atkin, 2015). Note how once again cognition is explicated as a certain kind of embodied action, making Peirce’s view also very congenial to enactivism.^15^ Of course, individuals can and do often disagree in their judgements, but Peirce stipulates that regularities are real insofar as all cognizers *would* eventually agree on them. He challenges us to articulate what we mean by the truth, if we do not mean this. In this way, for Peirce, like Zahidi, *reality is not token-mind-dependent, but it is type-mind-dependent* (Peirce CP: 5.430–2) – dependent on communities of inquirers. His account too is a form of internal realism.

Again, traditional realists may be troubled by the worry that type mind-dependence is still mind-dependence. *Qua* embodied action, is not inquiry subject to numerous vagaries, such as ‘lost facts’^16^ and error-states which remain forever unresolved? And does this not compromise our pragmatist realism on Brock and Mares’ second criterion? In reply we can say two things. Firstly, Peirce constructs a fresh form of objectivism by carving a space inside the ostensibly ‘mind-dependent’ for a newly-defined form of ‘mind-independence’. He says, “reality is independent, not necessarily of thought in general, but only of what you or I or any finite number of [persons] may think about it….” (Peirce CP: 5.408). This means that although Peircean reality is mind-dependent in the sense that it must be cognizable – there are no things in themselves – it is mind-*in*dependent in the sense that the community of inquiry always in principle retains the potential for further discovery of error. As Legg observes, “agreement amongst inquirers constitutes truth, but agreement amongst no cardinality of inquirers guarantees truth” (Legg, 2014a: 212). Peirce notes that this indefinite limit to his community of inquiry represents “the idea of fallibilism objectified” (Peirce CP: 1.171).

Secondly, we may now note that for Peirce the type-mind-dependence of *reality* does not entail the type-mind-dependence of *existence*. We have seen that Peirce defines existence in terms of the material world’s direct ‘imposition’ on us. Such ‘brute’ encounters notoriously outrun our understanding and anticipation of them. Peirce illustrates this in a famous example whereby “walking in the street reflecting upon how everything is the pure distillate of Reason, a man carrying a heavy pole suddenly pokes you in the small of the back”. After such an experience, he humorously concludes, “you may think there is something in the Universe that pure reason [i.e. Thirdness] fails to account for” (Peirce CP: 5.91-2). Thus, for Peirce, although the world’s cognitionary or intelligible character is type-mind-dependent, its dynamic character is manifestly not – its Otherness may surprise and shock us. At the same time, such Otherness provides the necessary spur to further inquiry which renders a subject’s world more intelligible through further discovery of real regularities. In this way, then, Secondness and Thirdness represent complementary modes of being, and pragmatic realism can satisfy Brock and Mares’ (2007) independence claim whilst still being compatible with Zahidi’s (2014) view of reality as type-mind-dependent.^17^ Surprising encounters with Peircean Secondness constitute such a rich source of learning that it is vital to accommodate them in any scientific realism. Yet insofar as Zahidi restricts reality to what subjects can *successfully* manipulate, he arguably fails to recognize the existence and value of these encounters, thereby weakening his realism. In Peircean categorical terms, we might say that Zahidi’s realism fails to do justice to both Thirdness qua intelligibility, and Secondness qua unsuccessful manipulation. Therefore we believe that we must move from a *manipulation-based*, to an *inquiry-based* realism. To this end, the next section will further explore Peirce’s pragmatic understanding of inquiry and its consonance with enactivist approaches to the mind. After that, in Section 6, we will discuss how under pragmatic realism, multiple subjects can be understood to cognize one single world.

## Pragmatic realism: cognition as inquiry

We have seen how, like Hacking and Zahidi, Peirce understands reality by reference to a certain kind of *action*, however the action he chooses to focus on is *inquiry*. We will now explore this connection further. Peirce’s epistemology requires more than mere causal manipulations of existent objects in particular contexts. It also requires continuous generalizing reinterpretations of experience. This is clearly seen in Peirce’s account of perception, which he understands to encompass two temporal directions simultaneously. In the first, backward-looking direction, new objects are perceived by means of a set of *preestablished habits*, developed through the subject’s previous interactions with the world. For example, if I perceive an apple, one possibility available to me is the action of eating, which has habitually satiated my hunger in the past, which disposes me to continue the habit. Meanwhile, in the second, forward-looking direction, a subject’s perception expresses a set of *future expectations* about how the environment will be experienced if the perception is accurate. For example, from the perceptual judgment “That is a red apple”, I expect that if I were to bite it, it would taste delicious.^18^ What Peirce took as a defining feature of his pragmatism – and expressed in his famous Pragmatic Maxim, in his article “How to Make Our Ideas Clear” (CP 5.388) – was that these past habits and future expectations constitute *the entire meanings of our concepts*.

Now, our expectations will or will not be actually met in the world. If they are met, our initial perception will be confirmed, and we will continue to have similar expectations, but if they are not met, we must adjust our expectations. For instance, imagine that I am hungry, perceive an apple in my office, and expect it to provide nourishment. I bite it and discover that it is plastic. Such an unpleasant surprise will motivate me to change how I perceive and interact with apple-looking objects in future. I will reinterpret my initial perception, which I understood to be of a delicious apple, as in fact being of a hard and tasteless object, in hope that I can avoid inadvertently biting plastic items in future. This reinterpretation of my previous perception will ramify through my habits and expectations in ways that are impossible to predict in advance. It is not overstatement to describe this re-interpretation as a kind of world re-construction. It is reminiscent of enactivist Varela’s claim, cited above, that it is not possible to “understand our world as inseparable from our minds’ own processes of self-modification” (Varela et al., 1991: 139).

We noted how Peirce identified the real with what a community of inquirers would agree on at the limit of inquiry. Let us now consider this claim more fully. As a preliminary remark, note that Peirce’s definition does not imply that intersubjective agreement is necessary to determine what is real, or sufficient at any given point in time, only that understanding the notion of reality involves understanding the *possibility* of agreement in a community of inquirers. (Reality does not consist only in what is cognized, though it must be cogniz*able*.) It follows that inquiry is not a human-only activity, nor does it require the ability to use language or logical reasoning. The crucial notion is *agreement*, which simply refers to the possibility of subjects *aligning their lived expectations*. For example, faced with an unknown object, a human and a cat might both be prompted to interact with it. If it is experienced as painfully hot, both creatures will form new expectations about future interactions with it. If it moves in their direction, both will likely move away. Here we can say that the two creatures have reached nonlinguistic *pragmatic agreement* about the object.

Accordingly, it is important to note that the reference in Peirce’s definition to a *community of inquirers* simply refers to the actual or possible subjects who could in principle reach pragmatic agreement with respect to a given object. (This is despite customary use of the term ‘inquiry’ to denote funded research projects and the like. In Peirce’s sense the concept is much more naturalistic than sociological.) It is also worth noting that in defining reality by means of pragmatic agreement, we need not *actually* unite all possible inquirers, but rather we can consider whether they *would* have the same set of expectations if they were interacting with a given object. This ‘would-be’ clause is in fact what renders Peirce’s view a form of realism, rather than the varieties of instrumentalism or conventionalism that are often associated with pragmatism.^19^ The so-called “end of inquiry”, is not “a description of some future time where all questions are settled” (Legg, 2014a: 206), but an idealised continuation of subjects’ current activities of working towards pragmatic agreement by developing and revising their expectations of the world. Such developments and revisions form new habits of action that may be gradually coordinated through ongoing interaction and coupling with both the environment and other cognizing subjects. We will now explore how this possibility for coordination enables our view to *unify the worlds of all knowing subjects* – not in the metaphysical sense that is usually taken for granted in philosophical discussions of realism, but *as an epistemic achievement across time*.

## Pragmatic realism: securing a single world

We now return to the key problem for Zahidi’s realism – that by constructing a world from what subject-types can successfully *manipulate*, his view leaves no room in principle for explaining how subjects who do not interact with the same entities can cognize the same world. Nor can scientists working within his realist framework explain how differing cognitive perspectives arise from differing embodiments interacting with one underlying world. Our view addresses these two issues.

So far we have claimed that under our pragmatic realism, X is real if it would generate pragmatic agreement over the long run in a community of inquirers of indefinite scope. But the Peircean framework also enables us to posit more circumscribed communities of inquiry in light of more specific debates. For instance, we might make a claim such as this:

X is an overlapping feature of two different realities R_1_ and R_2_ of species S_1_ and S_2_, if X would generate pragmatic agreement in case enough inquiry about X was carried out by members of S_1_ and S_2_.

This ability to posit more specific communities of inquiry enables us to *ask a much more nuanced series of questions about realism*. For instance, we might ask whether X is real with respect to:

- **(1)** one species
- **(2)** some set of species
- **(3)** all possible species.

It is vital to be clear about which question we are asking when we argue about realism, as these three questions will often deliver different answers in specific contexts. A great deal of previous philosophical discussion about realism regarding phenomena such as color has arguably suffered through lacking this kind of clarity. For instance, because humans are trichromatic, the range of colors they can see is different from that of tetrachromatic creatures such as pigeons. Call H the color reality that would be pragmatically agreed on by humans, and P the color reality that would be pragmatically agreed by pigeons. As pigeons are able to see ultra-violet plumage, because it helps with mate selection, ultra-violet colors are real features of P, but not H. But we should not infer from this that pigeons inhabit a different world than humans, or that we now need to be anti-realists about color. The claim that ultra-violet colors are real features of P offers an answer to question **(1)**, not **(2)** or **(3)**. We might say that these colors are *real for pigeons*, although not for humans. On the other hand, if we ask which features of the world are commonly perceived by both humans and pigeons (question **(2)**), the answer will be different.^20^

We can then take one step further and ask question **(3)**: what are ‘the’ real colors perceivable by all species?^21^ This question is what most philosophers generally *assume* that they have in mind when they discuss color realism,^22^ reflecting in part the attempt of modern philosophers to describe reality as what is cognizable from what Nagel (1989) calls a “view from nowhere”. As Chirimuuta (2017, p. 15) notes, this assumption lies at the center of many disagreements between realists and anti-realists about color, where well-argued answers to questions such as **(1)** and **(2)** arguably often sneak in and muddy debates that ostensibly concern **(3)**. If we give up the idea that **(3)** is the only way to frame realism questions, as Chirimuuta believes we should, a pragmatic understanding of reality in relation to specific communities of inquiry can be of great benefit – both in signalling the existence of a more nuanced series of questions, and in delineating the contours of the questions themselves.

Note that we are not saying that question **(3)** is not important. Such broad challenges have frequently proven crucial for scientific progress. Peirce himself urges that although more local realism questions have an extremely important role to play at waypoints along the ‘road of inquiry’, they should ultimately be placed within a monist framework which postulates a single overarching reality which would be recognized by any cognizing being in the sufficiently long run. Already in 1878, he explicitly stated that this would generate a non-anthropomorphic, pan-species concept of reality:

Our perversity and that of others may indefinitely postpone the settlement of opinion; it might even conceivably cause an arbitrary proposition to be universally accepted as long as the human race should last. Yet even that would not change the nature of the belief, which alone could be the result of investigation carried sufficiently far; and if, after the extinction of our race, another should arise with faculties and disposition for investigation, that true opinion must be the one which they would ultimately come to (Peirce CP: 5.408).

As noted above, this one shared reality should not be regarded as a metaphysical posit so much as an epistemic achievement across time, by cognizing subjects of all species, insofar as they do succeed in aligning their habits and expectations. Of course, it is impossible to prove that such a state of affairs is achievable. Peirce increasingly acknowledged this towards the end of his career. Yet he maintained that as a *regulative hope*, his pan-species realism spurs us towards valuable scientific inquiry. For instance, within the framework of Peirce’s realism it *is* possible to frame the question that we argued Zahidi’s realism left no room for: the explanation of *how* particular sensori-motor embodiments (*qua* cognitive ‘perspectives’) give rise to specific kinds of experiences – for instance, the look and feel of trichromatic as opposed to tetrachromatic vision.^23^

It must be acknowledged that enactivism’s original pioneers explicitly repudiate pan-species realism, as an attempted reinstatement of the foundationalism that they have worked so hard to transcend. They argue against understanding enactive cognition as evolutionary *adaptation*, because it promulgates “the idea that organisms are basically parachuted into a pregiven environment” (Varela et al., 1991: 198). Instead, they propose a model of *natural drift*, whereby different species follow cognitive pathways constituted by unique mind-environment couplings that are equally “viable”, and as such, “incommensurable” (Varela et al., 1991: 201). They thereby assume that it is impossible to unify trichromatic and tetrachromatic sensoria in a fuller understanding of experience of which both human and pigeon color spaces are intelligible parts, and we should not try. But how can the authors of *The Embodied Mind* really know that this is impossible? It is one thing to express scepticism that such a feat might be achieved, but to construct a theory in which it is impossible in principle is quite another. Here we urge that these early enactivists are committing the cardinal sin that Peirce referred to as ‘blocking the road of inquiry’ (calling it “the one unpardonable offence in reasoning” Peirce CP: 1.136). Urging us against this offence is, we believe, another way in which Peirce’s scientific realism is powerful and as yet underappreciated.

These considerations finally put us in a position to see why the association between enactivism and anti-realism discussed in Section 2 is too hasty. From a pragmatic realist perspective, where enactivist arguments against realism have gone wrong is in assuming that recognizing the type-mind-dependence of the *reality* of the world—its intelligible or cognizable character—should force us to conceive of its *existence*—its reactionary character—also in mind-dependent terms. In other words, the reluctance of some enactivists to accept that there is a mind-independent world shared by multiple species results from a conflation of the notions of existence and reality.

This conflation has, in turn, motivated the idea, implicit in enactivist arguments against realism, that saying that the world’s existence is mind-independent commits one to the view that there is just *one* way in which that world can be cognized or made intelligible—that the existence of the world implies that its reality is “pregiven” (Varela et al., 1991: 198). Unsurprisingly, this idea is difficult—if not impossible—to square with a central tenet of enactivism, which is that there is a “world of lived experience” whose structure is characterized and delimited by factors other than the world’s existence—that is, the structural coupling relations between organisms and the world. But by distinguishing between existence and reality and by denying that the existence of the world fixes its reality, pragmatic realism makes room for a view in which the *reality* of the variety of worlds of lived experience, determined by structural coupling relations, can be preserved without requiring that we deny the *existence* of a single world that is shared by multiple species.

## Conclusion

We began by noting that enactivism is a fascinating recent addition to philosophy of mind, which problematizes the metaphysical separation between mind and world posited by many traditional realisms. We have shown that although enactivism might seem *prima facie* incompatible with realism – and the original enactivists explicitly abandoned realism in favour of a so-called ‘middle way’ between it and idealism – this move is not necessary. Understanding realism through Brock and Mares’ Existence and Independence Claims, we first examined Zahidi’s attempt to reconcile it with enactivism, by constructing a world from that which subject-types can successfully manipulate. We found that in its focus on manipulability rather than intelligibility, this account multiplies worlds to the point where it is no longer possible to explain *how* specific features of different epistemic perspectives might be created by different lived embodiments, thereby putting unnecessary limits on scientific inquiry. We then turned to Peirce, exploring his distinction between existence and reality and his inquiry-based theory of cognition, in order to show how it resolves these issues.

Although pragmatism has been widely associated with anti-realism, at least since James (1975), Peirce famously disputed this, even coining a new term for his view – *pragmaticism –* which he hoped was sufficiently uninviting to dissuade imitators. The key to understanding Peirce’s distinctive realism is to understand what a pragmatic account of reality amounts to – the habits and expectations which would be converged on by any cognizing subjects, given sufficient experience. The state of pragmatic agreement at the ‘end’ of inquiry should not be understood as an actual temporal moment that is yet to be achieved (see Hookway, 2004; Legg, 2014a). Rather, this account of reality simply generalizes from the countless ways in which we *do* continuously find out new things about the world. Pragmatic realism is therefore not an instrumentalism, for it does not hold that reality is what is pragmatically agreed at any given time, nor is it determined by the practices of any given group of subjects. At the same time, through its recognition of the existence and role of relatively stable ‘cognizer-types’ (be they communities of inquiry in the human world, or species in the non-human world), the Peircean framework enables us to distinguish a nuanced set of questions concerning whether X is real *for a given cognizer-type* – where traditional realism can only treat X as real or unreal *simpliciter*. This is valuable as cognitive science matures to embrace greater recognition of non-human animal cognition. As such, pragmatic realism should be attractive for traditional realists not only because it preserves their insights, but also because it shows the way towards integrating realism with important new developments in cognitive science. Finally, although a lineage from pragmatism to enactivism has been acknowledged by many enactivists (Gallagher, 2017; Hutto & Myin, 2012; Menary, 2016) these authors have tended to look to Dewey instead of Peirce for inspiration.^24^ Thus this study arguably addresses an important gap in enactivist literature.

## Data Availability

N/A.

## Footnotes

1 The realism we favour bears comparison to Hasok Chang’s recent book in defence of a pragmatist-inspired realism (Chang, 2022), which also argues that the concepts of truth and reality need to be brought back to the realm of practice and what scientists *do* (as opposed to mainstream views that construe truth and reality purely in terms of the possession of information). Where our view differs from Chang’s is in the extent to which his construal of the goal of inquiry as ‘operational coherence’ favours here-and-now accommodations, and thereby encourages pluralism about truth. Our more future-directed Peircean realism, by contrast, leaves space for a ‘realistic monism’.

2 See for instance (Wheeler, 1997; Thompson, 2010). It should be noted that (Ward et al., 2017) attempts to corral the Mind-Life Continuity Thesis into the earliest, so-called “autopoietic” branch of enactivism, arguing that it has no purchase on later, so-called “sensory-motor” (Noë, 2004) and “radical” branches (Hutto & Myin, 2012, 2017). We disagree that the Thesis is not held by sensory-motor enactivists. We concede the point for radical enactivism, and hope to explore this issue further in future work. We are grateful to an anonymous referee for pressing us on this.

3 A referee rightly points out that some of these ideas have been central to philosophical traditions such as classical pragmatism, of which Peirce is a central figure. However, until recently these traditions have not been viewed as mainstream.

4 Some theorists of embodied cognition theorise ‘action-oriented representations’ (e.g. Clark 2016; 2015; Wheeler, 1997). On the other hand, the ‘radical enactivism’ of Hutto and Myin treats anti-representationalism as defining of enactivism (Hutto & Myin, 2012, 2017). For a helpful overview of these ‘varieties of enactivism’, we again recommend (Ward et al., 2017).

5 See also (Miller, 2016) for a similar conceptual analysis.

6 All numbered citations of Peirce’s work refer to his Collected Papers (Peirce 1931-1958), indicating volume and paragraph, respectively.

7 This association goes back to (Varela et al., 1991), where the founders of enactivism raised the question of realism and idealism as it pertains to enactivism (drawing on Buddhist ideas), explicitly denying realism in favour of an enigmatic middle position between it and idealism.

8 We note here that the term “world” is often used ambiguously in discussions about enactivism and realism. According to one understanding, “world” refers to the lived experience that is enacted by subjects and determined by structural coupling relations. Alternatively, “world” is sometimes understood as referring to a spatiotemporal ‘container’ that organisms and objects inhabit, whose structure is independent of any one of those organisms and objects. We see the tension between enactivism and realism as motivated in part by this ambiguity. In other words, given that the latter understanding of “world” is what realists typically have in mind, it is difficult to see how it could be reconciled with the idea that there is a world of lived experience that is in any way mind-dependent. Our argument in the rest of the paper can thus be read as an attempt to show how these two understandings of “world” are not necessarily at odds. We return to this issue in Section 6.

9 Since Chemero’s proposal focuses on realism in scientific practice, we here focus on Zahidi’s, which attempts to extend entity realism to ordinary objects of cognition, such as tables and trees.

10 For the curious, these would be the self-described representatives of realism in mainstream analytic philosophy – figures such as David Lewis and Michael Devitt, and more recently, Ted Sider and Jonathan Schaffer.

11 Of course, the suggestion here is not that the contents of the floorplan need to be known beforehand to explain the differences in perspectives, but only that we need to *posit* the existence of a floorplan whose contents *would* explain those differences. This is precisely why we prefer an inquiry-based over a manipulation-based realism. We expand on these points in Sections 5 and 6.

12 We suspect that this restriction to pure manipulability and reactivity is why Hacking’s entity realism – whilst issuing a needed correction to philosophy of science, which previously neglected this dimension of reality – still represents a minority position within it.

13 It should be conceded that Zahidi does envisage some kind of feedback loop whereby learning through experience takes place, as objects are manipulated (Zahidi, 2014: 471, n13). But he does not really explain how such generalised learning is possible under his account, if cognition is *exhausted* by manipulation, as he appears to claim.

14 This is by no means the only kind of Thirdness, but it is the kind that we will focus on here.

15 For an extended exploration of Peirce’s philosophy of cognition as a variety of enactivism, with particular reference to the role played by his semiotics (sign theory) in building an embodied account of meaning, see (Legg, 2021).

16 One author offers as an example: “the number of cakes on a particular tray at a specific time during a party held years ago” (Johnston, 1993: 91).

17 Importantly, we are not suggesting here that traditional realists will readily endorse the Peircean account, but only that if they decide to reject it, their motivation will need to be other than that the view does not accommodate Brock and Mares’ definition of reality.

18 For invaluable scholarship on Peirce’s philosophy of perception, see (Rosenthal, 1994, 2004). For more recent work, see (Legg, 2014b, 2017; Wilson, 2017; Sant’Anna, 2018).

19 Here, we note, the difference between our view and the pragmatist realism presented in (Chang, 2022) really comes to the fore.

20 Here we do not mean to claim that the set of features that count as real for a certain species is always fixed. If humans were placed in an environment in which seeing tetrachromatic colors increased their fitness, they might eventually evolve the power to perceive tetrachromatic colors, at which point such colors would become ‘real for humans’. This fits with the point made above about perception’s ‘forward-looking’ aspect, and how it prompts subjects to continually re-align their expectations to their environment.

21 We should also acknowledge the possibility that at some stage in future science colour predicates might no longer play any role at all, as noted by Sellars in his discussion of the ‘manifest’ and the ‘scientific’ image (Sellars, 1963).

22 For a classic formulation, see (Armstrong, 1961: 161-4).

23 Peirce signalled that his philosophy aimed to address this question in a 1902 outline of a future science of “Psychics”, organized into “Nomological psychics”, which “discovers the general elements and laws of mental phenomena”, “Classificatory psychics”, which “classifies products of mind and endeavors to explain them on psychological principles”, and “Descriptive psychics”, which “endeavors in the first place to describe individual manifestations of mind” (Peirce CP: 1.189).

24 For further discussion of the implications of this significant omission, see (Fanaya, 2021; Legg & Reynolds, 2022).
